# Pregnancy in Women with Complex Congenital Heart Disease. A Constant Challenge

**DOI:** 10.5935/abc.20190197

**Published:** 2019-12

**Authors:** Walkiria Samuel Avila, Veronica Martins Ribeiro, Eduardo Giusti Rossi, Maria Angelica Binotto, Maria Rita Bortolotto, Carolina Testa, Rossana Francisco, Ludhmilla Abraão Hajjar, Nana Miura

**Affiliations:** 1 Instituto de Coração do Departamento de Cardiopneumologia da Faculdade de Medicina da Universidade de São Paulo, São Paulo, SP - Brazil; 2 Clinica Obstétrica do Departamento de Obstetricia e Ginecologia da Faculdade de Medicina da Universidade de São Paulo, São Paulo, SP - Brazil

**Keywords:** Pregnancy, Heart Defects, Congenital/complications Maternal Mortality, Fetal Mortality, Maternal and Fetal Outcomes

## Abstract

**Background:**

The improvement in surgical techniques has contributed to an increasing number of childbearing women with complex congenital heart disease (CCC). However, adequate counseling about pregnancy in this situation is uncertain, due to a wide variety of residual cardiac lesions.

**Objectives:**

To evaluate fetal and maternal outcomes in pregnant women with CCC and to analyze the predictive variables of prognosis.

**Methods:**

During 10 years we followed 435 consecutive pregnancies in patients (pts) with congenital heart disease. Among of them, we selected 42 pregnancies in 40 (mean age of 25.5 ± 4.5 years) pts with CCC, who had been advised against pregnancy. The distribution of underlying cardiac lesions were: D-Transposition of the great arteries, pulmonary atresia, tricuspid atresia, single ventricle, double-outlet ventricle and truncus arteriosus. The surgical procedures performed before gestation were: Fontan, Jatene, Rastelli, Senning, Mustard and other surgical techniques, including Blalock, Taussing, and Glenn. Eight (20,0%) pts did not have previous surgery. Nineteen 19 (47.5%) pts had hypoxemia. The clinical follow-up protocol included oxygen saturation recording, hemoglobin and hematocrit values; medication adjustment to pregnancy, anticoagulation use, when necessary, and hospitalization from 28 weeks, in severe cases. The statistical significance level considered was p < 0.05.

**Results:**

Only seventeen (40.5%) pregnancies had maternal and fetal uneventful courses. There were 13 (30.9%) maternal complications, two (4.7%) maternal deaths due to hemorrhage pos-partum and severe pre-eclampsia, both of them in women with hypoxemia. There were 7 (16.6%) stillbirths and 17 (40.5%) premature babies. Congenital heart disease was identified in two (4.1%) infants. Maternal and fetal complications were higher (p < 0.05) in women with hypoxemia.

**Conclusions:**

Pregnancy in women with CCC was associated to high maternal and offspring risks. Hypoxemia was a predictive variable of poor maternal and fetal outcomes. Women with CCC should be advised against pregnancy, even when treated in specialized care centers.

## Introduction

In the last decade, the continuous and progressive improvement in the surgical and late postoperative treatment has allowed an increased number of children with complex congenital heart disease (CCC) to reach childbearing age.

The registry of 1000 cases of pregnant women followed at the Heart Institute of São Paulo (InCor) ^1^ between 1989 and 1999 showed that during that 10-year period, congenital heart diseases corresponded to 19.2% of the cases, which represented the second most frequent structural cardiac lesion. Among them, less than 1% were congenital complex lesions.

However, over the last 50 years, there has been a significant tendency in our country to an increase in the percentage of CCC during pregnancy, as observed throughout the world, as shown by the European Registry for Cardiac Diseases in Pregnancy ( ROPAC) , in which 20% of 66% of pts with congenital heart disease had complex heart defects.^2^ This setting required a risk stratification scheme to predict adverse outcomes in pregnant women with congenital heart diseases seeking guidance to conceive. The modified World Health Organization (WHO) ^3-4^ classification is the most well-accepted risk stratification model for pregnancy in congenital heart disease patients, and it considers CCC as risk III, which means medical advice against pregnancy.

Despite this advice, both desired and unplanned pregnancy rates have been gradually rising, thus increasing the number of pregnant women with CCC. The scarcity of publications on pregnancy evolution in these women motivated this study.

## Objectives

Evaluate the evolution of complex congenital heart disease patients during pregnancy and identify variables related to poor maternal and fetal outcomes.

## Method

This is an observational and retrospective study of 435 pregnant women with congenital heart disease, consecutively included in the InCor-Registry of Pregnancy and Cardiac Disease during a period of 10 years (2007 to 2017). Among of them, 42 pregnancies in 40 congenital heart disease patients, classified as complex by Bethesda conference^5,6^ (Table 1) and included in risk category III by WHO^3,4^ (Table 2) were selected for this study.

**Table 1:** Types of Adult Patients with Congenital Heart Disease of Great Complexity

Conduits, valved or nonvalved
Cyanotic congenital heart disease (all forms)
Double-outlet ventricle
Eisenmenger's syndrome
Fontan procedure
Mitral atresia; Tricuspid atresia; Pulmonary atresia
Single ventricle (also called double inlet or outlet, common or primitive)
Pulmonary vascular obstructive diseases
Transposition of the great arteries
Truncus arteriosus/hemitruncus
Other abnormalities of atrioventricular or ventriculoarterial connection, not included above( crisscross heart, isomerism, heterotaxy syndromes, ventricular inversion)

Warnes AC et al. J AM Coll Cardiol 2001, 37:1161

**Tabela 2:** Modified World Health Organization (WHO) classification of maternal risk during pregnancy in congenital heart disease

WHO I	Risk no higher of maternal mortality and very low morbidity: uncomplicated, small or mild (pulmonary stenosis; ventricular septal defect; patent ductus); successfully repaired simple lesions (atrial or ventricular septal defect; patent ductus arteriosus; anomalous pulmonary venous drainage).
WHO II	Small increased risk of maternal mortality and morbidity: unoperated atrial or ventricular septal, repaired tetralogy of Fallot, repaired coarctation, atrioventricular septal defect.
WHO III	Significant increased risk of maternal mortality and morbidity: systemic right ventricle (e.g. congenitally corrected transposition, simple transposition after Mustard or Senning repair); Fontan circulation ; cyanotic heart disease; other complex congenital heart disease
WHO IV	Very higher risk of maternal mortality and morbidity or severe morbidity Pulmonary arterial hypertension of any cause; severe systemic ventricular dysfunction; heart failure and LVEF <30%; severe left heart obstruction, severe (re)coarctation; Fontan with any complication.

WHO: modified World Health Organization; LVEF: left ventricular ejection fraction

During the first prenatal appointment, all the patients had anatomical and functional diagnosis defined by the InCor-Congenital Heart Disease Team and began periodic follow-up every two weeks until the second trimester of pregnancy. Subsequently, this follow-up was altered to a consultation every week, during the third trimester, with the same cardiologists and obstetric teams according to the protocol established by the InCor- Pregnancy and Heart Disease Team. The protocol included:

- Advice on general measures (rest, restricted physical activities, control of anemia and eventual infections, dose adjustment or substitution of drugs to adapt to the current pregnancy status) ;
- Periodic evaluation of the oxygen saturation, maternal hematocrit and hemoglobin;
- Assessment by congenital heart diseases specialists (including new echocardiographic study);
- Follow-up with obstetrical team;
- Elective hospitalization of high-risk patients after 28 weeks (hypoxemia, pulmonary hypertension, serious obstructive lesions and important ventricular dysfunction) and
- Delivery according to obstetrical indications;
- Infective endocarditis prophylaxis during delivery with intravenous Ampicillin 2.0 g associated to gentamicin 1.5 gr/kg/intramuscular, applied one hour before delivery;
- Postpartum appointment for clinical check-up and collection of information on the delivery, based on the clinical summary of the hospital discharge, as well as on the maternal and newborn complications.

The following maternal variables were considered for this study: age, baseline heart disease; prior heart surgery; hypoxemia (oxygen saturation< 92% at rest, measured by digital oximeter and/or clinical signs of peripheral cyanosis) ; maternal hematocrit and hemoglobin; ventricular dysfunction (ventricular ejection fraction (EF) ≤ 50%) ; occurrence of heart complications, obstetric complications and maternal death.

Regarding the newborn, the variables considered were: gestational age at the delivery, fetal loss classified as: miscarriage (< 20 weeks), stillbirth (between 20 and 36 weeks) and neonatal death (up to 30 days after delivery) and malformations related to the maternal heart disease.

The conditions: hypoxemia, prior heart surgery and univentricular anatomy were studied as predictive variables of maternal and fetal outcomes.

### Statistical analysis

The categorical variables were considered in the tables containing absolute (n) and relative (%) frequencies. The association of the categorical data was evaluated using the chi-square method and, when necessary, Fisher's exact test. The distribution of the quantitative variables regarding normality was evaluated with the Kolmogorov-Smirnov test. The variables with normal distribution were presented as mean and standard deviation and compared using Student’s *t*-test for independent samples. The non-parametric variables were presented as median and interquartile interval and compared using the Mann-Whitney test. The values of p < 0.5 were considered significant. The *SPSS* software version 18.0 was used for the statistic calculations.

This research was approved by the Institutional Review Board of Hospital das Clínicas of the School of Medicine of the University of São Paulo - SDC protocol 4563/17/063.

## Results

The baseline clinical characteristics of 40 patients at the beginning of pregnancy and the types of structural cardiac lesions and previous surgical repairs, obstetric and fetal outcomes of the 42 pregnancies are shown in tables 3 and 4, respectively.

**Table 3:** Baseline characteristics of 40 pregnant women

Clinical status	
Age (years), mean ± dp	16 to 41 (mean 24.5 ± 3.4)
Oxygen saturation (%)	76 - 99 (mean 88.5)
Hemoglobin (mg/dL)	10,5 - 22,0 (mean 14.8)
Hematocrit (%)	32 - 69 (mean 47)
Functional Class (NYHA) (%)	I and II: 35 (79%) pts
	III: 9 (21%) pts
	IV: 0 (0%) pts
Previous surgical repair	34 (77.3%) pts
No previous surgical repair	8 (20%) pts
Hypoxemia (Sat% < 92%)	19 (47.5%) pts

Dp: standard deviation; NYHA: New York Heart Association; Sat %: oxygen saturation (measured by digital oximeter); Pt: patient.

**Table 4:** The baseline of cardiac defects, types of previous surgical repair, obstetrical and fetal outcomes: 42 pregnancies

Case	Age (Years)	Specific Cardiac Defects	Surgical repair technique	Sat %	Hb/Ht	Delivery/ Gestational Age/ Newborn Weight/gr
1	18	d-TGA	Senning	99%	13.5/ 39	Vd/ 37/2150
2	20	AP + VSD	Fontan	80%	19.7/ 62	Cs/29/1950
3	21	AP + VSD + APC	no repair	80%	15.0 / 44	Vd/27/1750
4	18	Truncus arteriosus	Rastelli	99%	12.5/38	Cs/40/3360
5	20	AP + VSD + PDA	Rastelli	98%	11.6/ 35	Cs/33/1860
6	19	d-TGA	Jatene	96%	11.2/ 33	Vd/37/2230
7	33	DVSVD + PS	Conduit LV- Ao + RV - PT	98%	12.8/35	Cs/38/2850
8	27	DVEVE + AAVC	no repair	93%	15.9/47	Vd/37/2630
9	18	d-TGA + VSD + PS	Jatene	98%	12.1/ 36	Vd/38/3886
10	32	d-TGA + PS + VSD	BT + Rastelli	98%	10.5/ 32	Vd/38/3210
11	24	DVSVD+dTGA+ PS+VSD	Conduit VE-AO + Rastelli	99%	11.5 / 36	Vd/37/2250
12	19	dTGA+ASD+VSD+ PS	no repair	80%	17.0/54	Miscarriage
13	32	PA	BT + Glenn	76%	17.5/50	Cs/36/1980
14	23	dTGA + VSD	Jatene	87%	12.5/40	Cs/34/2130
15	21	Single Ventricle	Glenn+ Fontan	92%	13.5/42	Cs/31/1400
16	29	DVSVD	BT + Glenn	78%	13.8/49	Cs/29/1250
17	38	Single ventricle	Fontan	82%	16.8/50	Miscarriage
18	27	Single ventricle	Fontan	83%	16.9/50	Cs/28/850g
19	19	TA + VSD + PS	BT + Glenn +Fontan	87%	15.0/47	Vd/37/2030
20	29	PA + VSD	Rastelli + Conduit RV-PT	90%	13.0/42	Cs/37/2350
21	32	AT+ASD+ VSD + PS	Fontan	94%	10.4/32	Cs/35/1800
22	34	Single ventricle	no repair	89%	22.0/69	Abortion
23	29	d-TGA	Senning	93%	11.9/40	Vd/ 39/ 2720
24	30	DVEVE + VSD	Pulmonary truncus banding	89%	17.0/49	Cs/35/1750
25	16	d-TGA	Jatene	96%	12.5/40	Cs/38/3420
26	22	AT	no repair	85%	13.7/40	Cs/32/1150
27	29	d-TGA	Jatene	93%	12.2/39	Cs/38/2460
28	32	DVSVD+d-TGA+PS	Mustard	93%	12.5/45	Cs/36/2240
29	40	d-TGA	Jatene	95%	12.3/42	Cs/37/2570
30	17	DVEV único-E+ ASD+ VSD+PS	no repair	93%	15.9/47	Vd/ 39/ 2720
31	22	DVEV único -E	no repair	94%	12.8/45	Vd/37/2630
32	24	Truncus arteriosus	Conduit valved	93%	12.5/42	Cs/38/3270
33	32	dTGA+ ASD +VSD+ PS	Fontan	91%	13.0/43	Vd/33/1510
34	26	AP + VSD	no repair	87%	21.0/67	Vd/30/1120
35	18	dTGA+ASD+VSD+PS.	Senning	90%	11.8/42	Vd/38/2770
36	19	d-TGA + VSD+ASD+ PS	Conduit LV + Reconstruction atrioventricular	93%	11.8/42	Cs/37/2410
37	20	d-TGA	Jatene	95%	11.9/40	Cs/38/3220
38	41	AP + VSD+PDA	BT + Collateral arterial repair	87%	15.4/45	Cs/28/500
39	31	DVSVD + VSD	Rastelli + Conduit LV-AO	96%	13.4/40	Vd/36/2480
40	1	d-TGA +VSD+PS	Rastelli+ Conduit LV-Ao	90%	11.5/40	Vd/38/3500
41	27	AT + VSD + PS	BT+ Glenn + Fontan	93%	13.8/40	Cs/34/1750
42	24	Truncus arteriosus	Ao + VSD repair + pulmonary graft	94%	11.5/40	Cs/37/2168

TA: tricuspid atresia; ASD: interatrial communication; VSD: interventricular septal defect; AP: pulmonary atresia; D- TGA: complete transposition of the great arteries; DVEVE: double inlet left ventricle; DVSVD: double outlet right ventricle; AAVC: abnormalities of atrioventricular or ventriculoarterial connection; PS: pulmonary stenosis (infundibular,valvar or supravalvar); AP: pulmonary atresia; APC: aortopulmonary collateral arteries; PDA: patent ductus arteriosus; Truncus: Persistent truncus arteriosus; LV: left ventricle; Ao: aorta; RV: Right ventricle; PT: pulmonary truncus; BT: Blalock Taussing procedure; Sat %: oxygen saturation; HT: hematocrit % ; HB: hemoglobin mg/dl; Vd: vaginal delivery; Cs: cesarean section.

The analysis of the structural or functional cardiac lesion recorded at the beginning of the pregnancy (table 4) showed: hypoplastic right ventricle in cases 2, 16, 20, 23 and 26; left ventricular dysfunction (EF < 50%) in cases 11 and 20; valvular, infundibular or supravalvular stenosis, with gradient > 50 mmHg in cases 14, 20, 24, 25, 26, 30, 31, 35 and 37; important valvular regurgitation in cases 17, 19, 27, 28 and 32. Eight (20.0%) patients were unoperated. The anatomical and functional analysis showed that 16 (40%) patients were considered as univentricular hearts.

### Maternal and fetal outcomes (Figure 1)

**Figure 1:**
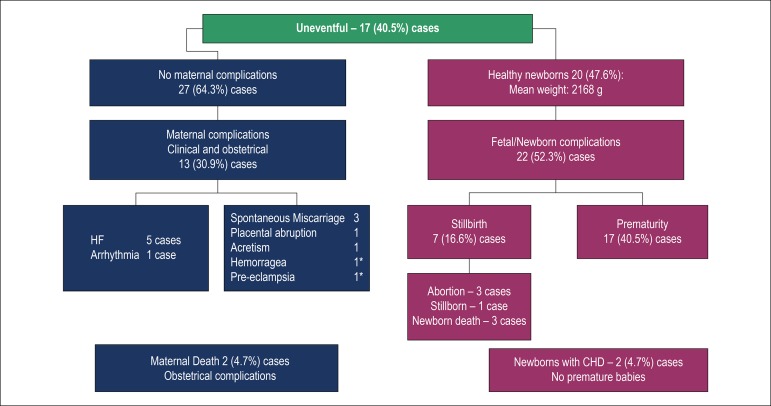
Maternal and fetal outcomes: 42 pregnancies. HF: heart failure; Acretism: placental acretism; CHD: congenital heart disease.

Maternal and fetal success was considered in 17 (40.5%) cases, when the mother and healthy newborn were discharged from the hospital after delivery without complications. Figure 1shows the maternal-fetal evolution and complications. Heart failure occurred in cases 5, 6, 15, 24 and 41 (Table 4) and was treated with hospitalization, strict hygiene-dietetic measures, furosemide, carvedilol or metoprolol associated or not to digitalis, when indicated. Electrical cardioversion was needed for atrial flutter treatment in case 21 (Table 4). The hospitalization duration for the treatment of complications or for childbirth planning varied between 21 and 68 days (average of 45 days). There were two maternal deaths (4.7%) related to obstetric complications: hemorrhage after delivery and preeclampsia, cases 34 and 38, respectively (Table 4).

The obstetric complications are shown in Figure 1. The fetal losses correspond to miscarriages in cases 12, 17 and 37, stillbirth in case 34 and neonatal death in cases 14, 16 (premature babies) and 35 (Table 4). The delivery occurred on average at 37 weeks of gestation; 24 (54.5%) were Caesarian section due to obstetric raisons or progressive maternal clinical worsening. Among the live newborns, there were two (4.7%) cases of congenital heart disease: one with recurrence of maternal heart disease (case 25) and the other with tetralogy of Fallot (case 32) ; neither of the cases were preterm babies.

Among of the predictive variables of maternal and fetal outcomes, hypoxemia showed a significant correlation with worse pregnancy prognosis, while prior surgery (whether the mother had been submitted to previous surgery or not) and univentricular function showed no correlation with the maternal and fetal outcomes (Table 5).

**Table 5:** Comparative analysis of presumptive variables of maternal and fetal outcomes

Variables No cases	Hypoxemia n = 19	No hipoxemia n = 23	p value	Univentricular n = 16	Bi-ventricular (n = 26)	p value	Operated n = 34	Unoperated n = 8	p value
Unventfull evolution n = 17	3 (15.7%)	14 (60.8%)	< 0.05	4 (25%)	13 (50%)	0.0	14 (41.2%)	3 (37.5%)	1.0
Maternal complications n = 13	9 (47.4%)	4 (17.4%)	< 0.05	7 (43.7%)	6 (23.1%)	0.18	10 (29.4%)	3 (37.5%)	0.68
Maternal death n = 2	2 (10.5%)	0	0.19	0	2 (7.7%)	0.51	1 (2.9%)	1 (12.5%)	0.34
Fetal complications n = 23	18 (94.7%)	5 (21.7%)	< 0.05	12 (75%)	11 (42.3%)	0.06	18 (52.9%)	5 (62.5%)	0.70
Stillbirths n = 7	7 (36.8%)	0	< 0.05	4 (25%)	3 (11.5%)	0.39	3 (8.8%)	4 (50%)	< 0.05
Premature babies n = 17	12 (63.1%)	5 (21.7%)	< 0.0.5	10 (62.5%)	7 (26.9%)	< 0.05	14 (41.2%)	3 (37.5%)	1.0
Mean weight newborns (g)	± 600	± 527	< 0.05	1841 ± 454	2531 ± 496	< 0,05	2246 ± 660	2296 ± 749	0.76

## Discussion

This study included one substancial series of pregnant CCC patients, submitted to the multidisciplinary protocol at the Heart Disease and Pregnancy tertiary care center. This studied group represented 9.6% of 435 pregnancies in women with congenital heart diseases included in the InCor-Registry during the last decade. It is undeniable that higher post-operative survival of these patients will result in an increasing number of pregnancies in women with CCC in the near future.

The CCC considered in this study were included in the WHO risk category III,^3,4^ which means pregnancy is discouraged, justified by the rates of 25.5% of maternal complications and 70% of poor fetal outcome. These considerations are according to the results of this study, which recorded only 40% of successful pregnancies, i.e., healthy mothers and newborns without complications. The high rates of maternal events (36%) and fetal events (43%) are the bases for the WHO guideline that advises against pregnancy in this group of patients.

However, the global experience in this clinical situation is increasing and it represents a major challenge for clinicians. Occasionally, women become pregnant without prior counselling or sometimes they desire a pregnancy despite the advice against it.^7^ The diversity of the anatomical and functional conditions of the heart defects in CCC restrict the creation of management protocols for eventual complications during pregnancy, delivery and postpartum.^7^

However, knowledge of the most common complications that occur in the late postoperative period of CCC helps in the management of pregnancy in these patients. In this regard, a study about the causes of death in patients with CCC showed that heart failure, sudden death, ischemic heart disease and infective endocarditis were the most common ones. In addition, the most significant anatomical lesions (except for Eisenmenger's syndrome) were the transposition of the great arteries and the Fontan circulation.^8^

A study of 120 necropsies of congenital heart disease patients^9^ confirmed heart failure as the main cause of death, since the ventricular remodeling in response to volumetric and pressure overload during life favors fibrosis, hypertrophy and a reduced number of myocardial interstitial capillaries. Thromboembolism, which is the second cause of death, was significantly associated with histological signs of pulmonary hypertension, also detected in chronic hypoxemia. The third cause of death recorded was infective endocarditis, which reinforced the recommendation of antibiotic prophylaxis during delivery in our protocol.

The routine elective hospitalization as of the 28^th^ week of pregnancy for patients at likely higher risk situation, regardless of the functional condition, was based on the fact that the third trimester is critical for the mother, due to the hemodynamic overload and higher prothrombotic activity, as well as for the fetus, due to the high incidence of prematurity and intrauterine growth restriction, which are characteristics of CCC.

Furthermore, elective hospitalization improved maternal and fetal monitoring, allowed intermittent oxygen therapy to be applied, individualized anticoagulation, and optimized therapy for possible complications and delivery planning.

Our results showed similar rates of cardiac (14.2%) and obstetric complications (16.6%). In both cases, maternal deaths were associated with obstetric causes (pre-eclampsia and postpartum hemorrhage). This result allows a reassessment of the severe cardiac reserve limitation in these patients, who do not tolerate the events inherent to pregnancy and postpartum, regardless of the baseline functional condition.

Pre-eclampsia, one of the causes of death in this study, is responsible for 15% of maternal deaths in Brazil, with an incidence of around 10% in the pregnant population.^10^ Early diagnosis and an effective prenatal care, although they do not prevent the disease, can improve maternal mortality in healthy women. However, in patients with complex heart disease, the prognosis of pre-eclampsia is much worse, due to both systemic endothelial dysfunction, inherent to the disease and the circulatory overload caused by arterial hypertension.

Postpartum hemorrhage, the reason for the second death in our study, is considered an important obstetric cause of maternal death in women with heart disease, especially in hypoxemic patients. This fact was documented in a study of 366 primiparous women with congenital heart diseases, which recorded 21% of postpartum hemorrhage. That study identified early Caesarean section, general anesthesia and use of low-molecular-weight heparin at both prophylactic and therapeutic doses, as variables of higher correlation with postpartum bleeding. Furthermore, women with Fontan circulation had the highest blood loss and the difference remained significant after correcting for the other variables.^11^

In our study, the association of hypoxemia and postpartum hemorrhage associated with Caesarian delivery resulted in the second maternal death. Actually, the highest maternal and fetal morbidities associated with premature Caesarean delivery is due to maternal clinical instability and intrauterine growth restriction, common in complex heart situations.

A brief emphasis should be placed on the most common heart disease in this cohort, such as the transposition of the great arteries and the Fontan circulation. The promising evolution after the correction of the transposition of the great arteries through atrial inversion (Senning’s procedure or Mustard’s technique) or arterial inversion (Jatene’s technique) has allowed the development of pregnancy.^12,13^ However, there are expected events in the late postoperative period that may be unfavorable to the success of pregnancy. Supraventricular arrhythmias and ventricular dysfunction in adulthood may occur in about 40% of patients after surgical correction using Mustard’s technique or Senning’s procedure. On the other hand, neo-aortic valve regurgitation, present in 1-2% and coronary complications observed in 3-11% of cases, may both occur in the long term after Jatene’s technique. Regarding Rastelli's surgery, the late evolution depends on the type of tissue used, which can determine different degrees of calcification and progressive occlusion of the graft.^14^

Based on these considerations, during pregnancy, one must be prepared for the treatment of heart failure and arrhythmias when there is right ventricular dysfunction and tricuspid regurgitation after Mustard’s and Senning’s techniques; heart failure and low output due to calcified conduits after the Rastelli procedure, of valve dysfunctions and/or coronary complications, when Jatene’s technique had been used.^14^

This study showed that, with the exception of one patient who was not operated whose pregnancy ended in miscarriage (case 12), the other pregnant women with transposition of the great vessels of the base showed favorable maternal and fetal evolutions, regardless of the type of surgical correction. It is worth emphasizing that the expected complications were controlled with hospitalization and constant monitoring of the mother and fetus.

Fontan circulation has allowed the survival of 70% of the patients with univentricular heart disease up to childbearing age.^15^ However, in the late postoperative period, complications such as atrial tachycardia, thromboembolism (related to hepatic and venous system stasis) , heart failure, liver failure and, protein-losing enteropathy. The abnormal connection - vena cava and pulmonary circulation - despite the improvement in cyanosis, ventricular overload reserve and pulmonary circulation capacity, may be *threatened* by the variations in the central venous pressure and by the negative intrathoracic pressure induced by hyperventilation and changes in cardiac output during pregnancy.

The inability of patients with Fontan circulation to adapt to physiology of the pregnancy and postpartum period was documented in our study that showed worsening of the functional class in all the patients. Heart failure occurs because the abnormal anatomical and functional ventricle is unable to adjust to the increased cardiac output. However, there was no maternal death due to a good responses were obtained to the clinical treatment with the use of diuretic and beta-blocker in patients who developed heart failure (cases 15 and 41), and to electric cardioversion in case of atrial flutter (case 21).

On the other hand, the evolution unfavorable to the fetus was documented in six Fontan cases, resulting in a miscarriage and five premature deliveries. Review of the literature that included six studies with 255 pregnancies and 133 women showed 137 (69%) fetal loss, and 68 (59%) of the 115 live newborns were premature and six (5,2%) evolved to neonatal death. The causes of prematurity were not detailed, particularly in those induced by the anticipation of the delivery due to maternal reasons. However, the premature rupture of the amniotic membranes and placental premature detachment occurred in 6.2% and 10.9%, respectively.^16^

The poor fetal prognosis was confirmed by the multicenter study of the United Kingdom that included 50 women, 124 pregnancies, showed an incidence of 68 (54.8%) miscarriages and, among the 56 (45.2%) live newborns, four died due to extreme prematurity (delivery with gestational age below 32 weeks). On the other hand, the maternal complications (heart failure in 13.5%, arrhythmias in 11.3% and pulmonary thromboembolism in 1.19% of the cases) do not result in maternal death.^17^

The full anticoagulation routine in patients with Fontan circulation should be considered by reasons of the high risk of thromboembolism peculiar to this setting and the hypercoagulable state of the pregnancy and postpartum. This present study showed three cases of maternal thromboembolism, two of which were in a non-anticoagulated patient.

Patients with Fontan circulation should advise against pregnancy specially in patients with depressed ventricular function, cyanosis, important mitral valve insufficiency or protein-losing enteropathy.^15-17^

This study showed that hypoxemia was the presumable variables of the worst maternal-fetal prognosis, as previous report.^18^ The unusual result of the previous surgical correction did not show any difference in the evolution of the pregnancy, possibly was related to a small number of the cases and some cases had cardiac lesion with an anatomy favorable to survival during childbearing age. Therefore, another study that analyzed 102 necropsies of congenital heart failure patients verified that the average age of cases not operated was higher than those operated and presented also less serious anatomical defects.^9^

Another highlight of our study is the record of two newborns (5%) with of congenital heart disease which equivalent to six times more than the 0.8% estimated for the general population. This rate is still above the value by Oliveira et al. reporter which identifies 3 (3.2%) of cardiac malformations in children from 100 pregnant women with congenital heart diseases followed at InCor.^19^ The recurrence of heart disease in the babies of mothers with congenital heart diseases should be considered in counselling before pregnancy in response to the questions about hereditary as well as indication of routine fetal echocardiography study.

### Study limitations

Both the small number of patients and the wide heterogeneity of the anatomical defects contribute to a limitation of the accurate statistical analysis. However, it should be considered that this sample of patients was exclusively from the high risk group, in which pregnancy is not advised and constitutes a great guidance dilemma regarding family planning provided to women with CCC. The character of the study (retrospective and observational, restricted to a single center) can also influence the appropriate conclusions.

### Final comments

Congenital heart diseases affect approximately 0.8% of all live newborns and the survival rate of 86% are highlighted in international records. It is estimated that there are currently more adults with congenital heart diseases than children, which naturally provides a considerable number of women at childbearing age.

The qualification of the multidisciplinary team is fundamental in the counselling of young women with heart disease regarding pregnancy, including advice such as alternatives to a safe and effective childbirth. Despite the stratification of the WHO risk III that allows the advice against pregnancy, the provision contained in the Brazilian Legislation should be considered:

*Furthermore, article 226 should be considered: Based on the principles of human dignity and responsible parenthood, family planning is a free decision made by the couple, and it is the State’s responsibility to provide educational and scientific resources to exercise this right, prohibiting any enforcement by official or private institutions” (our emphasis). This standard applies to other institutes: a) human dignity (article 1, III) and b) right to freedom (article 5, caput)*^20^

## Conclusions

The strict care protocol during pregnancy, delivery, and puerperium did not prevent maternal deaths, prematurity or miscarriage in patients with CCC. Hypoxemia was a poor prognostic factor and maternal evolution was unsatisfactory, but the fetal outcome was worse.. Although the autonomy of intention to conceive should be respected, women with CCC should still be advised against getting pregnant.
